# The Warburg effect as an adaptation of cancer cells to rapid fluctuations in energy demand

**DOI:** 10.1371/journal.pone.0185085

**Published:** 2017-09-18

**Authors:** Tamir Epstein, Robert A. Gatenby, Joel S. Brown

**Affiliations:** 1 Cancer Biology and Evolution Program, Moffitt Cancer Center, Tampa, FL, United States of America; 2 Department of Biological Sciences & Cancer Center, University of Illinois at Chicago, Chicago, IL, United States of America; Mayo Clinic Rochester, UNITED STATES

## Abstract

To maintain optimal fitness, a cell must balance the risk of inadequate energy reserve for response to a potentially fatal perturbation against the long-term cost of maintaining high concentrations of ATP to meet occasional spikes in demand. Here we apply a game theoretic approach to address the dynamics of energy production and expenditure in eukaryotic cells. Conventionally, glucose metabolism is viewed as a function of oxygen concentrations in which the more efficient oxidation of glucose to CO_2_ and H_2_O produces all or nearly all ATP except under hypoxic conditions when less efficient (2 ATP/ glucose vs. about 36ATP/glucose) anaerobic metabolism of glucose to lactic acid provides an emergency backup. We propose an alternative in which energy production is governed by the complex temporal and spatial dynamics of intracellular ATP demand. In the short term, a cell must provide energy for constant baseline needs but also maintain capacity to rapidly respond to fluxes in demand particularly due to external perturbations on the cell membrane. Similarly, longer-term dynamics require a trade-off between the cost of maintaining high metabolic capacity to meet uncommon spikes in demand versus the risk of unsuccessfully responding to threats or opportunities. Here we develop a model and computationally explore the cell’s optimal mix of glycolytic and oxidative capacity. We find the Warburg effect, high glycolytic metabolism even under normoxic conditions, is represents a metabolic strategy that allow cancer cells to optimally meet energy demands posed by stochastic or fluctuating tumor environments.

## Introduction

ATP is the primary energy source for mammalian cells and is produced primarily through oxidative or non-oxidative (glycolysis) metabolism of glucose. Oxidative phosphorylation produces up to 36 ATP per mole of glucose while glycolysis results in just 2 ATP [1]. Hence, conventional models of cellular energy dynamics assume that oxygen availability determines the optimal ATP-producing metabolic pathway so that less-efficient glycolysis serves primarily as a reserve metabolism for periods of hypoxia [2].

Yet, cancer cells, as well as a variety of normal cells, frequently exhibit high rates of glycolysis even in the presence of normal oxygen concentrations. This is described as aerobic glycolysis and, in cancer, often termed the “Warburg effect” after Otto Warburg who first observed it almost 100 years ago [3]. Because aerobic glycolysis is inefficient, it maintains adequate energy supplies through increased glucose flux which can be imaged using F^18^ labeled deoxy-d-glucose and Positron Emission Tomography (FdG-PET). In the past decade, clinical application of FdG-PET has demonstrated that >90% of human cancers exhibit increased glucose uptake indicating aerobic glycolysis is a ubiquitous property of the malignant phenotype. This was recognized in the recent update of cancer hallmarks which now includes “energy dysregulation” [4].

Aerobic glycolysis is difficult to reconcile with the conventional model of carcinogenesis as “somatic evolution.” The inefficient production of ATP via anaerobic metabolism of glucose in the presence of oxygen seems inconsistent with maximization of cellular fitness that should follow from Darwinian dynamics. Presumably, natural selection in a resource-limited environment would strongly favor maximally efficient energy extraction from the limited supply of glucose. Though it was initially attributed to some sort of mitochondrial dysfunction, it is known today that most cancer cells retain functional mitochondrial metabolism and in some even increase [5]. This puzzle has defied explanation despite over 8 decades of investigation since Warburg’s initial 1929 observations.

**Fig 1:**
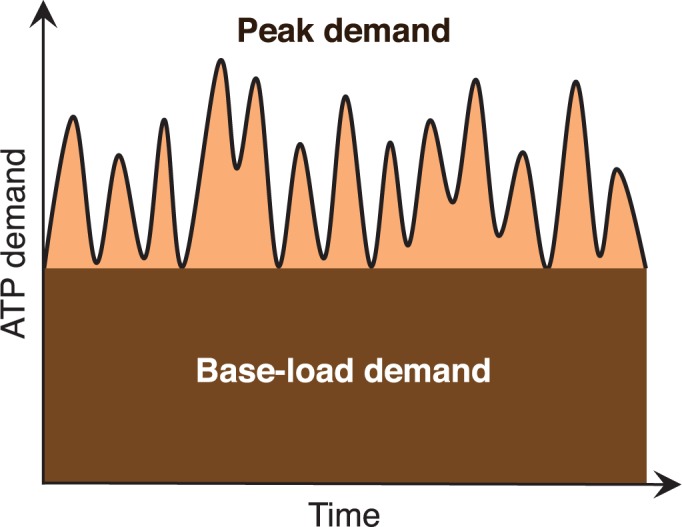
Schematic representation of the demand-driven metabolic model. ATP demand is composed of two types of demand; (dark) slow changing base-load demand, primarily for macromolecules synthesis and (light) peak demand, that rapidly changes primarily to support membrane transporters. In this model oxidative phosphorylation (high efficiency, slow response time) supplies base-load demand, and glycolytic metabolism (less efficient, fast response time) supplies peak ATP demand. The later is less efficient but is more highly responsive.

We have addressed this apparent paradox by developing an alternative model of glucose metabolism, in which the two metabolic pathways serve as complementary mechanisms for meeting ATP demands [6]. In our model (Fig 1), the speed of ATP production is balanced against efficiency. Oxidative phosphorylation, while yielding maximal numbers of ATP, is slow to respond to fluctuations in demand while glycolysis, though less efficient, can increase flux and ATP production far more quickly. When faced with temporally fluctuating needs for ATP, a tumor cell can optimize its energy production by maintaining a mix of metabolic capacities. We, thus, proposed that cells should use efficient but slow-responding aerobic metabolism to meet baseline, steady energy demands and glycolytic metabolism to meet short-timescale pulses in energy demands, primarily for membrane transport activities. Since almost every aspect of cancer development, including division [7], migration [8,9] and invasion [10], requires increased activity of membrane transporters, the Warburg effect is viewed as physiological response to large fluctuations in short-term energy demand that is necessary to maintain functions that are inherent in the malignant phenotype.

Here we broadly examine optimization of cellular energy production based on a tradeoff between efficiency and speed. While the efficiency of the two metabolic pathways is well studied, there is little information on the response time of glycolytic pathway in the literature. Available literature generally places the ratio of response times of these two pathways is about 100 [11]. That is, the glycolytic pathway can upregulate ATP production 100 fold faster that oxidative phosphorylation. However, it has also been hypothesized this ratio is actually higher due to allosteric regulation of glycolysis and limited diffusion of oxygen [12,13]. In our model simulations, we used ratios from *k*_*m*_ = 50 to 10,000

Interestingly, this energy management resembles the optimal operation of power grids in which different timescales of electricity demand are supplied by different types of power plants [14]. For example, efficient but expensive to build and maintain facilities, such as nuclear plants, are used to meet baseline demand while inefficient but fast responding gas turbines supply peak demand [15].

Here we apply a game theoretic approach to further examine our hypothesis that the Warburg effect emerges from the tumor cell’s need to balance speed and efficiency in energy production. A central assumption in our models is that tumor cells temporally exist in a temporally and spatially heterogeneous environment due to often chaotic blood flow within disordered intratumoral blood vessels. Thus, steep spatial gradients of substrate are common in in vivo cancer [16] as are cycles of normoxia and hypoxia so that both energy supply and demand may be unstable.

To examine the dynamical interactions between energy production and demand, we consider two scenarios. In the first, the cells must meet a temporally fluctuating demand for ATP. This fluctuating demand has a fixed baseline component with an additional cycling peak demand. For this scenario, the goal is to successfully generate the necessary ATP to meet demand at all times while maintaining the lowest metabolic cost to the cell. In the second scenario, the needs and opportunities for using ATP fluctuate stochastically. We make a distinction between production of ATP to maintain the cell survival, and ATP that permits maximum acquisition of additional substrate such as migration to regions of higher glucose concentration, or more rapid uptake during periods of glucose availability. Furthermore, we assume that tumor cells are subject to Darwinian dynamics so that each cell must compete for limited resources with other individuals within the local tumor population. Thus the optimal mix of metabolic pathways must balance the efficiency of baseline metabolism, the need for spare capacity to meet rare but potentially catastrophic events, the value of spare capacity to pre-emptively uptake resources following flushes of resources, and the costs associated with maintaining metabolic capacity. The model, for both scenarios, identifies when aerobic glycolysis is favored because it optimizes speed of energy production to ameliorate hazards and pursue opportunities.

## Results

### Model 1: The efficiency of multiple ATP production modes in varying energy demand

We consider the two modes of energy production: oxidative phosphorylation and glycolysis. Each has different efficiencies for ATP production and response times. To determine the optimal mix of energy production pathways, we first consider the metabolic capacity required to meet all energy demands. Then we calculate the cost of meeting these demands in terms of glucose consumption, damage from excessive levels of intra-cellular ATP, and the cost of maintaining the glycolytic enzymes. The goal is to identify optimal combination of energy strategies that minimize costs under variable environmental conditions. This model uses three coupled differential equations to model intracellular ATP concentrations, consumption, and production assuming variable demand and the two modes of ATP synthesis, glycolytic (*g*) and oxidative (*o*):

$$\frac{dU_{ATP}}{dt}=\overset{\mathrm{ATP}\;\mathrm{production}}{\overset{︷}{\left(c_{g}P_{g}+c_{o}P_{o}\right)\overset{\mathrm{ADP}\;\mathrm{availability}}{\overset{︷}{\left(1-U_{ATP}\right)}}}}-d\left(t\right)$$ (1)

$$\frac{dP_{g}}{dt}=k_{g}\left(1-\frac{U_{ATP}}{\left(1-U_{ATP}\right)sp_{g}}\right)$$ (2)

$$\frac{dP_{o}}{dt}=k_{o}\left(1-\frac{U_{ATP}}{\left(1-U_{ATP}\right)sp_{o}}\right)$$ (3)

Eq (1) describes the dynamics of ATP levels within the cell, *U*_*ATP*_. For convenience, the model normalizes this value to a range of between 0 and 1. The production of ATP is influenced by the overall capacity for glycolytic and oxidative metabolism, *c*_*g*_ and *c*_*o*_, respectively, and the rate at which a unit of each of these pathways can produce ATP, *P*_*g*_ and *P*_*o*_, respectively. The rate at which each unit of capacity can produce ATP is limited by the availability of ADP = (1-ATP). Noting this, the dynamics of these production rates, *P*_*g*_ and *P*_*o*_, can be written as declining functions of ATP. In our simulations the ATP level must exceed a minimum hold, U^*^_ATP_ to maintain cell viability [1]. We assume that at this level AMPK is activated as a stress response to low ATP level. The ATP demand term, *d*(*t*) = *BL*+*PL*(*t*) is the sum of a constant base-load demand, *BL*, and a peak-load term, *PL*(*t*), which fluctuates temporally. Detailed description on the construction of the fluctuating demand function is shown in S1 Appendix. Peak load may result from cyclical or unpredictable needs to pump excess metabolites out of the cell, external stressors to the cell that require active cell membrane responses, or the need to actively move from an increasingly depleted or less favorable spot to another more favorable place.

Eqs (2) and (3) describe the dynamics governing the rates at which glycolytic and oxidative capacity can supply ATP [17]. The first terms, *k*_*g/o*_, are the response times of the production modes, where we assume that *k*_g_>>*k*_o_. The response time of the glycolytic production mode is significantly larger than the oxidative mode. The terms in the brackets are logarithmic mass-action terms, which determine the change in the rate of the two production modes. Crucially, there are pathway specific set-points *sp*_*g/o*_. These set-points determine the ATP to ADP ratio at which the production rate will increase versus the ratio at which production rates will decrease. When ATP to ADP ratio equals the set-point value there will be no change in the production rate. Each pathways rate of change in production rate increases with its set-point. In our model, the cell manages its ATP supply and level by modulating the set-points. For a given base-load demand, in the absence of fluctuations, system will converge to a production rate that will maintain ATP to ADP ratio equal to the set point. To insure that there is no use of the less efficient glycolytic pathway under constant ATP demand, the optimal set point for glycolysis will always be smaller than the oxidative set point. We insured this by making *sp*_*g*_ = 0.995*sp*_*o*_. However, there will be some maximum rate of ATP production per unit of glycolytic capacity, *P*_*g*_^*max*^, and we use this value anytime Eq (2) results in a value above this. For convenience, and without loss of generality, we set the oxidative capacity to *c*_*o*_ = 1. In this way, the cell increases oxidative metabolism by raising *sp*_*g/o*_.

To determine the optimal investment by the tumor cell in glycolytic capacity, *c*_*g*_, we began by considering all combinations of *c*_*g*_ and *sp*_*g*_ that permit the cell to meet *PL* by insuring that $U_{ATP}\geq U_{ATP}^{*}$. To find a given combination, we seed the simulation with a given *c*_*g*_ and a starting value of $sp_{o}=U_{ATP}^{*}/\left(1-U_{ATP}^{*}\right)$. At the end of a run, we evaluate whether intra-cellular ATP ever dropped below the temporally fluctuating demand level. If $U_{ATP}<U_{ATP}^{*}$ at any given time, we increase the oxidative set point, *sp*_*o*_ (and as a result the glycolytic set point, *sp*_*g*_) until *U*_*ATP*_ remains above the threshold. Repeating this process, establishes the minimum oxidative set point, *sp*_*o*_, for each value of *c*_*g*_. This can be done for a range of base, *BL*, and peak, *PL*, ATP demands.

**Fig 2:**
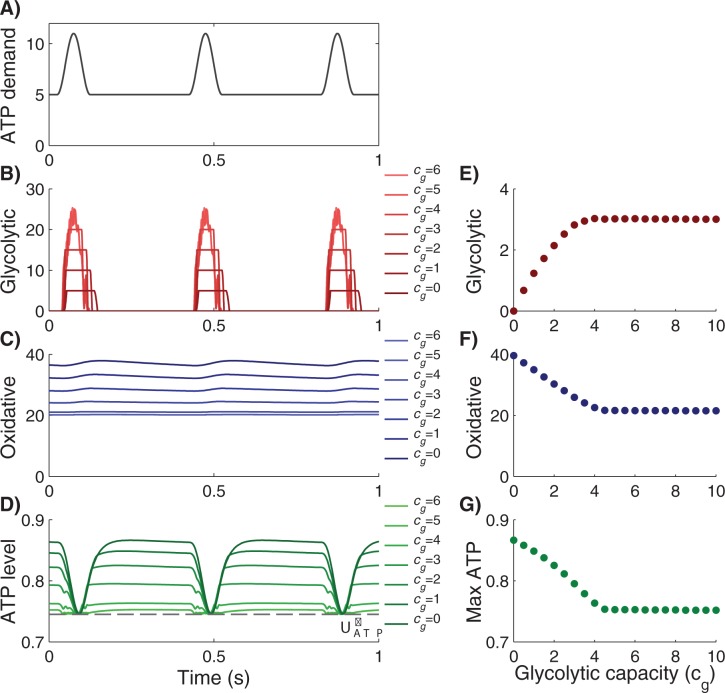
Typical dynamics of the system for different values of glycolytic capacity, *c*_*g*_. The optimal level of glycolytic capacity occurs where the curves in Panels E, F and G become flat. The left panels show the temporal dynamics of (A) ATP demand, *d*(*t*), (B) glycolytic ATP production, *c*_*g*_*P*_*g*_, (C) oxidative ATP production, *c*_*o*_*p*_*o*_, and (D) ATP level *U*_*ATP*_. Dash line denotes the minimum ATP level that is allowed in the system, U^*^_ATP_. Higher glycolytic capacity allows more glycolytic ATP to supply peak demand and reduces the maximum ATP level that is needed to ensure that *U*_*ATP*_≥U^*^_ATP_. The right panels show (E) total glycolytic ATP production over one period, *P*_*g*_^*total*^, (F) total oxidative ATP production over one period, *P*_*o*_^*total*^, and (G) maximum ATP level over the period max(*U*_*ATP*_). Parameters used in this simulation: Demand parameters: *f* = 10Hz, *period* = 4, *BL* = 5, *k*_*m*_ = 50, *k*_*g*_ = 10000, U^*^_ATP_ = 0.745, *P*_*g*_^*max*^ = 5 and *c*_*m*_ = 1.

Fig 2 shows typical dynamics of the system for different values of glycolytic capacity, *c*_*g*_. When there is no glycolytic ATP production (*c*_*g*_ = 0), oxidative phosphorylation must meet all ATP demands. Due to the long response time of the oxidative production mode, cells must maintain high concentrations of ATP at low ATP demand to avoid falling below the ATP threshold when peak-demand increases to its maximum. With the introduction of glycolytic capacity, some of the peak ATP demand is produced by glycolysis. However, this production can be no greater than *c*_*g*_ *P*_*g*_^*max*^. In this manner, low levels of glycolytic capacity will be run at maximum capacity, and so ATP production from glycolysis increases linearly with capacity. Eventually, glycolytic capacity, *c*_*g*_, reaches a level at which ATP production is no longer limited by the maximum ATP production per unit of glycolytic capacity, *P*_*g*_^*max*^. At this point, ATP from glycolysis no longer changes with capacity. We denote this capacity as the critical glycolytic capacity, c^*^_g_. At higher glycolytic capacity, there are no significant changes in the glycolytic and oxidative ATP productions and no change in the maximal ATP level. Any glycolytic capacity above this critical level is excess. Hence, this critical glycolytic capacity and associated oxidative set point, are the optimal metabolic pathways for a cell faced with fluctuating needs for ATP.

In these simulations the production cost (total cost over a complete cycle) is the sum of three terms:

π=−aA−cC−fF (4)

Where:

- $A=\max\left(U_{ATP}-U_{ATP}^{*}\right)$ is greatest harmful excess of ATP level in the cell during a fluctuation, and *a* is the per unit cost of that excess [18,19].
- *C* = *c*_*g*_ is the glycolytic capacity, and *c* is the per unit cost of having the glycolytic machinery [20,21].
- $F=f_{g/o}P_{g}^{total}+P_{o}^{total}$ is the amount of glucose consumed over a cycle, and *f* is the unit cost of glucose to the cell. Critical to total consumption of glucose is the production efficiency ratio, *f*_*g/o*_, of oxidative versus glycolytic ATP production. This ratio determines the additional burn rate of glucose via glycolysis to achieve the same ATP output as oxidation. If oxidation produces 6 times more ATP per glucose molecule than glycolysis then *f*_*g/o*_ = 6.

**Fig 3:**
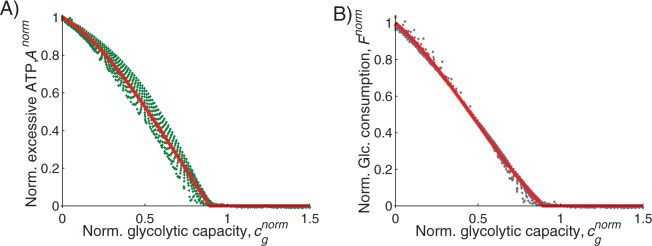
Normalized cost terms. (A) Normalized excessive ATP, *A*^*norm*^, and (B) normalized glucose consumption, *F*^*norm*^, as function of normalized glycolytic capacity, *c*_*g*_^*norm*^, for efficiency ration of *f*_*g/o*_ = 2. Dots are overlay of all values between *PL* = 1, 1¼, 1½, …, 5. Red lines are quadratic polynomial fit, which is used for calculating production cost for any value of peak-demand and glycolytic capacity. Whether presented as panel (A) or (B), the optimal level of glycolytic capacity that minimizes costs occurs at the point where normalized excess ATP or normalized glucose consumption reaches 0.

To simplify the calculations and the presentation of results, we normalize the glycolytic capacity by the critical capacity, c^*^_g_, and then by the magnitude of the peak demand, *PL*(*t*). Fig 3 shows the normalized excessive ATP term, *A*^*norm*^, and fuel consumption term, *F*^*norm*^. Normalization process payoff terms is detailed in the S2 Appendix. Thus, for a given peak-load magnitude and glycolytic capacity we can calculate the production cost by transforming to the normalized cost equation:

$$\pi^{norm}=-aA^{norm}-cC^{norm}-fF^{norm}$$ (5)

**Fig 4:**
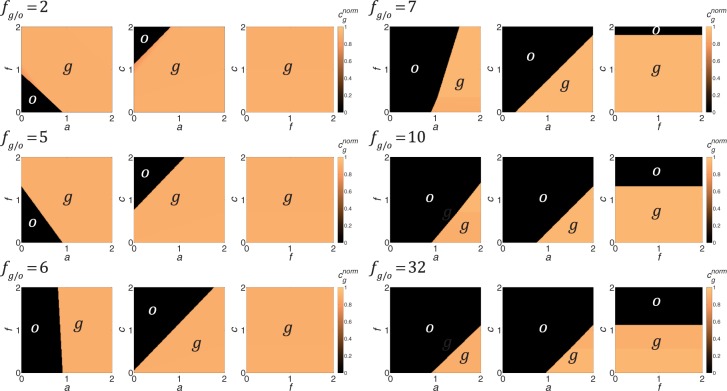
Optimal ATP production mode for peak-demand. The effects of the costs of excess ATP, *a*, glycolytic capacity, *c*, glucose, *f*, and the production efficiency ratio, *f*_*g/o*_, on whether it is optimal for the cell to use oxidative phosphorylation (*o*) or glycolysis (*g*) to meet peak ATP demand. As cost parameters change, there are abrupt shifts from one metabolic strategy to the other. The lines separating regions of oxidative versus glycolytic metabolism are called “isolegs” and their slopes and intercepts indicate how the cost parameters interact. Increasing the production efficiency ratio, *f*_*g/o*_, decreases the region over which glycolysis is optimal. Decreasing *a* or *c* generally favors glycolysis. The effect of *f* on the optimal metabolism for meeting peak demand depends on the relative glucose efficiency of oxidation versus glycolysis, *f*_*g/o*_. The values for all other parameters are the same as those in Fig 2.

Using Eq (5) we obtain the optimal proportion of glycolytic and oxidative ATP production, for any given peak-load magnitude, by calculating the normalized glycolytic capacity, *c*_*g*_^*norm*^, that minimizes the normalized production cost, *π*^*norm*^. The value of the optimal *c*_*g*_^*norm*^ indicates the peak demand that is produced by glycolysis, where *c*_*g*_^*norm*^ = 0 means all ATP comes from oxidative phosphorylation and at *c*_*g*_^*norm*^ = 0.85 about 99% of peak demand is supplied by glycolysis. Fig 4 demonstrates the results of these calculations for different values of the production efficiency ratio, *f*_*g/o*_ (higher value corresponds with less efficient glycolysis), and the three costs associated with excess ATP, *a*, maintaining glycolytic capacity, *c*, and unit cost of glucose, *f*. Each panel shows the optimal production mode for meeting peak demand as a function of the coefficient in the normalized production cost equation (Eq 5). As expected, oxidative phosphorylation is always the cheapest means for meeting base-load demand. A striking results concerns how peak demand is met. Rather than using a mix of metabolic pathways, it is almost always optimal to meet peak demand solely through one pathway or the other. The model shows that it is least costly to supply peak-demand either by glycolysis or by oxidative phosphorylation, but it is not profitable to supply peak demand by both production modes simultaneously. This can be attributed to concave shape of the glucose consumption payoff term and the excess ATP term (Fig 3).

Making the fuel demand of glycolysis less (decreasing *f*_*g/o*_) encourages the cell to meet essentially all of its peak demand through glycolysis (*c*_*g*_^*norm*^ = 0.85). When the glycolysis is 50% (*f*_*g/o*_ = 2) to 20% (*f*_*g/o*_ = 5) less efficient then for almost all parameter ranges peak load is met through glycolysis. For higher values of *f*_*g/o*_, the cell’s use of glycolysis depends strongly on the other cost parameters.

As would be expected, increasing the cost of having excess ATP, *a*, or decreasing the cost of maintaining glycolytic capacity, *c*, will encourage using glycolysis. One can consider all combinations of *a* and *c* such that the cell should switch from an oxidative to glycolytic metabolism for peak demand. In the state space of *a* (x-axis) and *c* (y-axis), this *a-c* isoleg (line of equal choice, [22,23]appears as a straight line with positive slope (Fig 4). The upper left corresponds to oxidative metabolism and the lower right to glycolysis. As the number of glucose molecules required to glycolytically producing the same number of ATPs as oxidatively increases, the isoleg retains roughly the same slope but the *c*-intercept declines. The intercept begins positive meaning that *c* = *a* = 0 favors peak-load glycolysis, and then becomes negative indicating oxidative phosphorylation is favored at *c* = *a* = 0. Hence, as *f*_*g/o*_ increases the region of glycolysis declines (Fig 4).

While it would seem intuitive that making glucose more available would favor glycoloysis, but the effect of the cost of glucose, *f*, is more nuanced and interacts strongly with the efficiency of oxidation relative to glycolosis, *f*_*g/o*_, and the cost of excess ATP, *a*. The slope of the *f-a* isoleg in the state space of *f* (y-axis) and *a* (x-axis) has negative slope that becomes increasingly vertical in going from *f*_*g/o*_ = 2 to *f*_*g/o*_ = 6. At *f*_*g/o*_ = 7 the slope becomes positive and near vertical and then the slope remains positive and declines at *f*_*g/o*_ = 10. The positive x-intercept of this isoleg remains roughly independent of *f*_*g/o*_. Hence, with increasing *f*_*g/o*_ the isoleg pivots from a positive to a negative slope in a clockwise direction (Fig 4). Regardless, to the left of isoleg is the region of oxidative metabolism and to the right is the region of using glycolysis to meet peak damand. When the isoleg has negative slope, increasing the cost of glucose can counter-intuitively shift metabolism from oxidative to glycolytic. When the isoleg has a positive slope, increasing the cost of glucose can shift optimal metabolism from glycolytic to oxidative.

The cost of glycolytic capacity, *c*, and the cost of glucose, *f* do not interact. The *c-f* isoleg in the state space of *c* (y-axis) and *f* (x-axis) is a horizontal line with a positive y-interecept. Above the isoleg, the optimal metabolism is oxidative and below glycoloysis is best for meeting peak ATP demand. The isoleg shifts to lower values of *c* as the glucose utilization efficiency of oxidation versus glycolysis, *f*_*g/o*_, increases. As expected, increasing the cost of a unit of glycolytic capacity can shift the cell metabolism from glycolysis to oxidative (Fig 4).

### Model 2: Glycolytic capacity

From model 1, cells should employ glycolysis to supply peak demand. However, in model 1, the magnitude of that peak demand was constant, and it occurred as a regular and predictable cycle. In reality energetic demand vary constantly and erratically. Uncertainty in the timing and magnitude of cellular ATP likely characterize the reality of most tumor cells. In our second model we assume stochastic fluctuations in energy demand, *d*(*t*). We distinguish between two types of energetic demands:

- Demands that are required to *exploit opportunities*, such as temporary fluxes of nutrients.
- Demands that are required to *avoid hazards*, such as changes in extracellular osmolarity.

While meeting the first type of demand increases the fitness of a cell, failure to supply the latter leads to fitness reductions. Meeting peak-demand requires glycolytic capacity, i.e. glycolytic enzymes. However, maintaining this capacity requires a fixed cost, which is linear with the capacity. Thus, our second model addresses the optimal capacity cells must maintain under uncertain peak-demand.

To find the optimal glycolytic capacity, *c*_*g*_, we used an evolutionary algorithm that represents a discrete version of Cohen’s evolutionary distributions [24–26]. We begin the simulations with 100 sub-populations each with a unique glycolytic capacity uniformly spread from *i* = 1 to 100. This gives a broad range of extant phenotypes among the cancer cells from which natural selection can “choose”. We let *x*_*i*_ denote the population size of each clonal subpopulation *i*. We use the following to describe the population dynamics of each discreet sub-population:

$$\frac{dx_{i}}{dt}=\left(\left(1+\alpha_{opp}D\left(t\right)\right)\left(1-\frac{\sum_{i}x_{i}}{x^{\max}}\right)-\alpha_{haz}S\left(t\right)+\alpha_{fix}c_{g_{i}}-m\right)x_{i}+\mathrm{drift}$$ (6)

$$\mathrm{drift}=\{\begin{array}{l} dx_{i-1}=dx_{i-1}+k_{drift}dx_{i}\\ dx_{i+1}=dx_{i+1}+k_{drift}dx_{i} \end{array}\;,\;dx_{i}=\left(1-2k_{drift}\right)dx_{i}$$ (7)

*α*_*opp*_, *α*_*haz*_ and *α*_*fix*_ are opportunistic, hazardous and fixed cost coefficients respectively. We also allow a small mutation rate. If a sub-population has a positive growth rate, *dx*_*i*_>0, then a fraction of that growth mutates into the phenotype of the adjacent sub-populations, *dx*_*i*-1_ and *dx*_*i*+1_ as shown in Eq 7. This represents an incremental mutation that either increase or decreases the individuals phenotype by one step.

For a given level of ATP demand, *d*(*t*), the supplied demand, *D*(*t*), and capacity shortage *S*(*t*), are:

$$D\left(t\right)=\{\begin{array}{ll} d\left(t\right) & d\left(t\right)\leq c_{g_{i}}\\ c_{g_{i}} & d\left(t\right)>c_{g_{i}} \end{array},\;S\left(t\right)=\{\begin{array}{ll} 0 & d\left(t\right)\leq c_{g_{i}}\\ d\left(t\right)-c_{g_{i}} & d\left(t\right)>c_{g_{i}} \end{array}$$ (8)

The entire population has a single carrying capacity so the different sub-populations negatively impact each other whether intra- or inter-population. All individuals interact ecologically. There are two sources of evolutionary change. The first represents changes in the frequency of each clad via different fitnesses, and the second comes from the mutations of individuals from one heritable strategy to another. The former dynamic favors the subpopulations with more successful glycolytic capacities, while the second creates variability in glycolytic capacity for natural selection to operate.

In the simulations we track the dynamics of each subpopulation in time (coupled ODEs equations) and fit the end-population to a Gaussian (S3 Appendix). To avoid stochastic artifact we repeated the simulations 30 times for each parameter.

**Fig 5:**
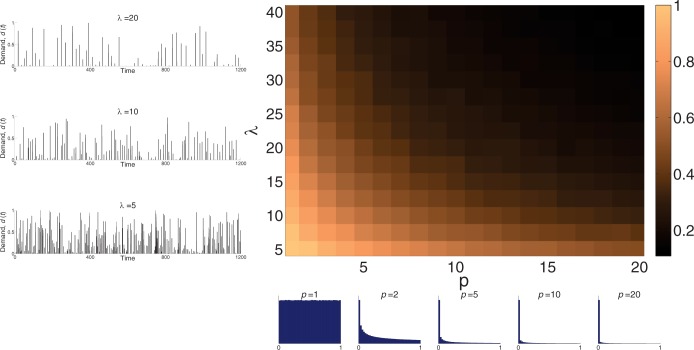
Influence of peak-demand parameters on the steady-state population distribution. Mean of population distribution as function power of the amplitude distribution, *p*, (bottom) and the mean time between events of demand, *λ* (left). The graph demonstrates that short time between bursts of peak-demand and high probability of high-amplitude demand results with a selection of population with high glycolytic capacity. Simulation parameters: *k*_*drift*_ = 0.05, *Α*_*haz*_ = 1, *Α*_*opp*_ = 1, *Α*_*fix*_ = 1 and *m* = 0.01. Detail description of the simulation process can be found in S3 Appendix.

We generated the peak-demand as spikes of demand with amplitude distribution of power law, *p*. The time between the demand spikes was Poisson distributed with parameter *λ*. Fig 5 illustrates the influence of these two parameters on the population distribution, showing the mean of the Gaussian function for the amplitude distribution, *p*, and the mean time between events of demand, *λ*. Increasing the power of the amplitude distribution and increasing the typical time between demands selects for low glycolytic capacity.

**Fig 6:**
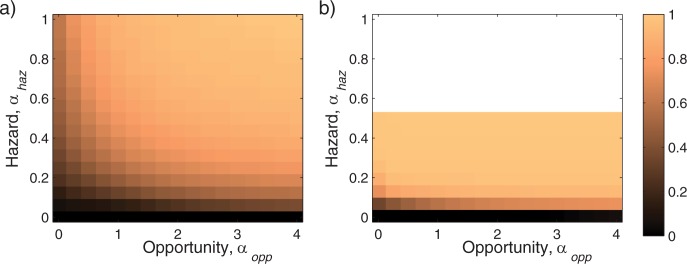
Influence of opportunistic and hazardous coefficient on the steady-state population distribution. Mean capacity that is selected as function of the opportunistic coefficient, *α*_*opp*_, and the hazardous coefficient, *α*_*haz*_ for fixed-cost coefficient, *α*_*fix*_ = 1 (a) and *α*_*fix*_ = 0.5 (b). The graphs demonstrate that hazard is more influential in selection of high glycolytic capacity than opportunity

The influence of opportunity and hazard on the population is presented in Fig 6, which shows mean population distribution as function of the opportunities coefficient, *α*_*opp*_, and the hazards coefficient, *α*_*haz*_. When opportunity and hazard events occur more frequently, selection for high glycolytic capacity is increased. Interestingly, at zero hazard, the optimal glycolytic capacity is almost zero, which was also observed when fixed-cost coefficient, *α*_*fix*_, was reduced by a factor of 2. This suggests that maintaining glycolytic capacity is more important for fending off hazards rather than exploiting opportunities.

## Discussion

The observation of high rates of aerobic glycolysis in cancer and some normal cells is inconsistent with the conventional model of glucose metabolism in which oxidative phosphorylation is viewed as the optimal pathway in all normoxic conditions. We introduced an alternative metabolic model in which glycolysis and oxidative phosphorylation are complementary modes of ATP production that trade-off efficiency and speed for meeting energetic demands. Oxidative phosphorylation is highly efficient in converting glucose to ATP but slow to responds to fluctuations in energy demand. Glycolysis is less efficient than oxidative phosphorylation but its response time is much greater than oxidative phosphorylation and, thus, well-suited to supply fluctuating demand, such as ion transporters on the cell membrane that are necessary for changes in the cell geometry during motion and invasion. In our model, optimal glucose metabolism is determined by both the availability of oxygen and the dynamics of energy demand.

Here we present two game-theoretic models that investigate optimal ATP-from-glucose production strategies for given energy demand conditions. In the first model we examined optimal ATP production strategies when cells experience periodic short-time increases in demand superimposed on a constant base-line demand. We note that this model is designed to study efficiency of ATP production for supplying short-term energy demand. As such, it does not address the interaction between the two pathways [27], such as initial ATP production by mitochondrial to support glycolytic metabolism [28]. Our results indicate the existence of two metabolic regimes, based on three critical parameters: the cost of excessive ATP level, the cost of uptaking glucose, and the cost of maintaining glycolytic capacity. We demonstrate that in normal, healthy tissue in which physiological conditions are spatially and temporally homogenous and demand fluctuations are small, it is optimal to produce all ATP by oxidative phosphorylation so that little or no glycolytic capacity is necessary. However, as the amplitude or frequency of demand spikes increases, the optimal metabolic strategy requires metabolic switching in which the constant component of ATP demand is supplied by oxidative phosphorylation and the fluctuating component by glycolysis (Fig 3).

The second model we investigate the trade-off between cost and benefit dynamics that governs the cell’s glycolytic capacity in typical cancer environments in which peak demand fluctuates due to alterations in local conditions caused by chaotic blood flow or host response. We studied two types of short-term ATP demands. The first one is generated by an opportunity to increase substrate acquisition in the event, for example, of a sudden increase in blood flow. We assume optimal response must be rapid because the opportunity diminishes with time either due to diffusion into adjacent tissue or consumption by other cells (scramble competition [29]). The second type is a demand that is required to avoid hazard (e.g. the sudden appearance of predator-like host anti-tumor T cells) and therefore must be met immediately to maintain survival. Our modeling results demonstrate that optimization of the glycolytic capacity includes an “acceptance” of the risk that a rare event provided there is long-term benefit to the population gained by reduction in the cost required to maintain sufficient energy capacity to overcome this rare threat. This results is supported by a recent study showing that adaptation to doxorubicin drug by expression of P-glycoprotein (PGP) transporters is followed by increase of glycolytic capacity[30], where the energy for these transporters is primarily supplied by glycolysis [6]. In contrast, there is little benefit in maintaining excess capacity to exploit uncommon spikes in environmental opportunities that would improve the energy status of the cell. From a broader perspective, our results demonstrate that “fear” is a stronger motivator for glycolytic capacity than “hunger”, which has been also observed in other ecological studies[31–33]. From a generic view this model address the fundamental economic question of capacity investment under uncertain demand [34–36], and therefore can be used in such studies.

In summary, our modeling results demonstrate that normal mammalian cells subject to a near constant environment will require limited glycolytic capacity. Normal cells that may be subject to frequent perturbations (on the skin or colon mucosa, for example) will probably have a higher capacity. Tumor cells in a benign, stable lesion (such as a fibroid) will likely have a low glycolytic capacity while cells in an invasive cancer with spatially and temporally heterogeneous blood flow and subject to immune attack, will likely need to maintain a high glycolytic capacity.

## Supporting information

S1 Appendix Construction of ATP demand function. (PDF)

S2 Appendix Normalization of payoff terms. (PDF)

S3 Appendix Glycolytic capacity simulations. (PDF)
