# Supplementary material for: The Warburg effect as an adaptation of cancer cells to rapid fluctuations in energy demand
Source: PLoS One. 2017 Sep 18;12(9):e0185085. doi: 10.1371/journal.pone.0185085 (PMC5602667; doi:10.1371/journal.pone.0185085)

S1 Appendix. Construction of ATP demand function

The demand function is based on the function:

(S1) $$d(t) = BL + Period\left\{PL\left(\sin\left(2\pi f\right)+1\right)\right\}$$

BL is base-load demand, PL is peak-demand and f is the frequency of peak demand. $Period$ is the periodicity of the peak-demand bursts, which is shown in the figure below.

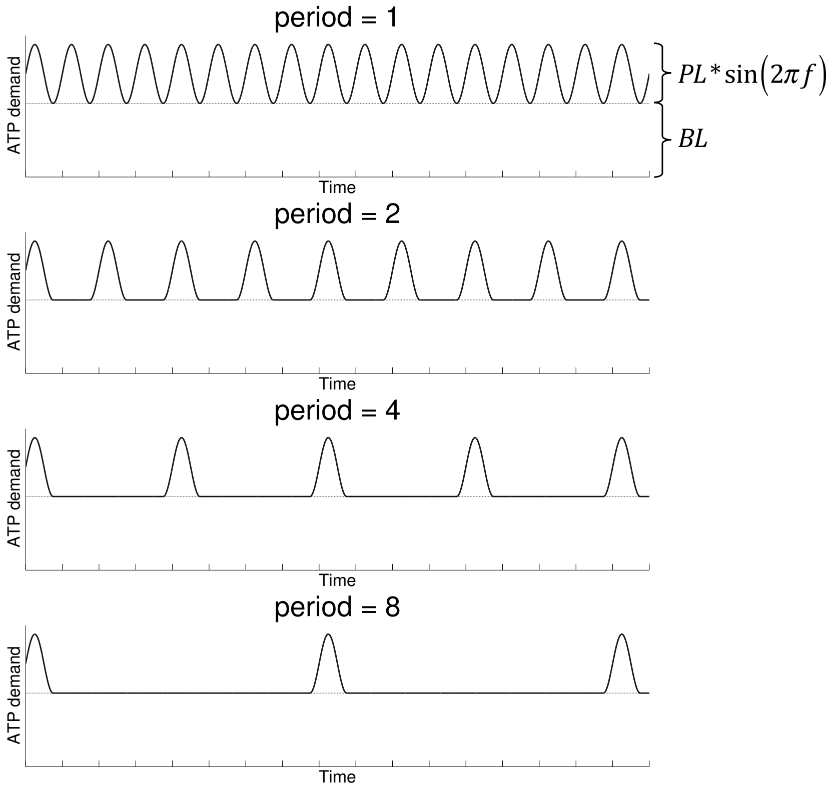

Supplement: S1 Appendix — (PDF) [file pone.0185085.s001.pdf]
