# Supplementary material for: The Warburg effect as an adaptation of cancer cells to rapid fluctuations in energy demand
Source: PLoS One. 2017 Sep 18;12(9):e0185085. doi: 10.1371/journal.pone.0185085 (PMC5602667; doi:10.1371/journal.pone.0185085)
Supplement: S2 Appendix — (PDF) [file pone.0185085.s002.pdf]

S2 Appendix. Normalization of payoff terms

The terms in the net-profit calculation depend on the glycolytic capacity and the peak-demand magnitude. To simplify net-profit calculations we normalized the payoff terms. Fig S2a shows the excessive ATP term, A, and glucose consumption term, F, as function of glycolytic capacity, c_g, for different peak-load magnitude values: PL=1,1¼, 1½, … ,5.

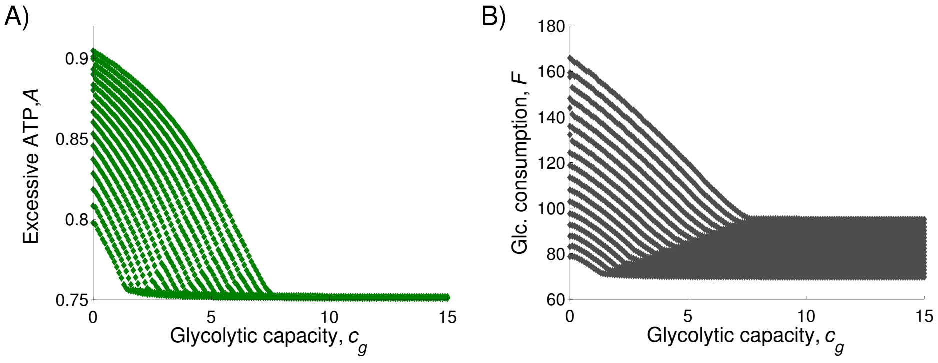

Fig S2a. Cost terms.

(A) Excessive ATP, A, and (B) glucose consumption, F, as function of glycolytic capacity, c_g, for efficiency ration of $f_{g/o}$=2, for peak level values of PL=1 ,1¼ , 1½, … , 5.

Our first step was to normalize the glycolytic capacity by linear fit of critical capacity, c_g*, and peak-load magnitude (as follows. The glycolytic capacity was normalized by the critical glycolytic capacity and the peak-demand (Fig S2b), obtaining:

$$c_g^{norm} = \frac{c_g}{1.62PL}$$

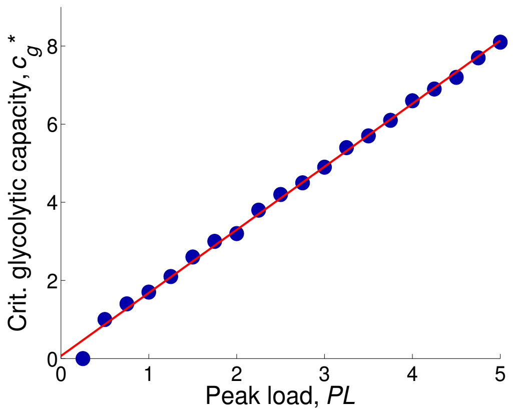

Fig S2b. Normalization of the glycolytic capacity.

Critical glycolytic capacity c_g*, as function of peak-demand magnitude *PL*.

Figure S2c depict the following steps in the normalization process, where the normalization terms are:

$$A^{norm}\left(PL\right)=\frac{A-0.756}{-0.0046PL^2+0.055PL+0.0094},\quad F^{norm}\left(PL\right)=\frac{F-6.029PL-0.01}{0.0949PL^2+10.39PL-3.444}$$

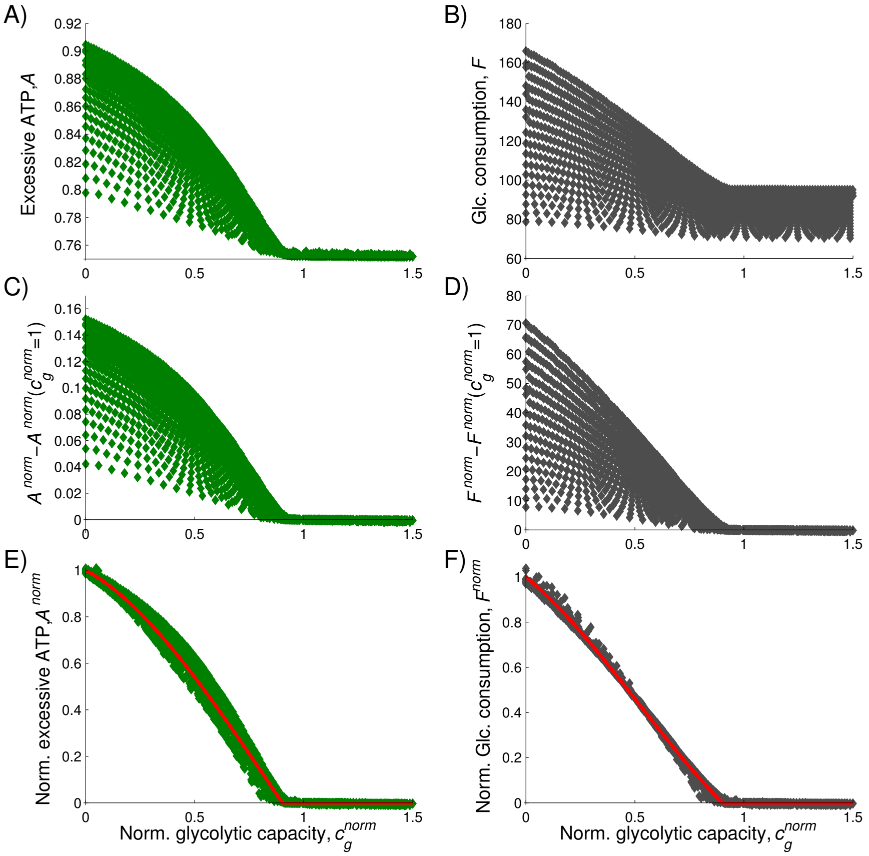

Fig S2c. Normalization process.

(A) Excessive ATP, *A*, and (B) glucose consumption, *F*, are subtracted by their value at c_g^{norm}=1 (C and D respectively), then divided by their value at c_g^{norm}=0 (E and F respectively). Red lines are quadratic polynomial fit.

The normalized cost values were fitted to a quadratic polynom obtaining:

$$F^{norm} = \begin{cases} -0.96\left(c_g^{norm}\right)^2 + 10.28c_g^{norm} - 3.23 & F^{norm} \geq 0 \\ 0 & F^{norm} < 0 \end{cases}$$

$$A^{norm} = \begin{cases} 0.0045\left(c_g^{norm}\right)^2 + 0.054c_g^{norm} - 0.0078 & A^{norm} \geq 0 \\ 0 & A^{norm} < 0 \end{cases}$$
