# Supplementary material for: The Warburg effect as an adaptation of cancer cells to rapid fluctuations in energy demand
Source: PLoS One. 2017 Sep 18;12(9):e0185085. doi: 10.1371/journal.pone.0185085 (PMC5602667; doi:10.1371/journal.pone.0185085)
Supplement: S3 Appendix — (PDF) [file pone.0185085.s003.pdf]

S3 Appendix. Glycolytic capacity simulations

We generated the peak-demand as spikes of demand with amplitude distribution of power law, *p*. The time between the demand spikes was Poisson distributed with parameter λ.

Dependence on amplitude distribution:

$\lambda = 10, p = 1$

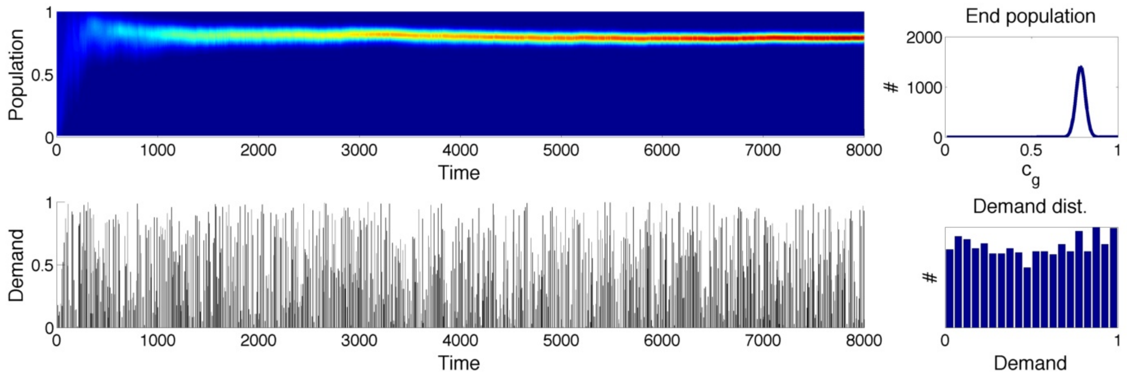

$\lambda = 10, p = 2$

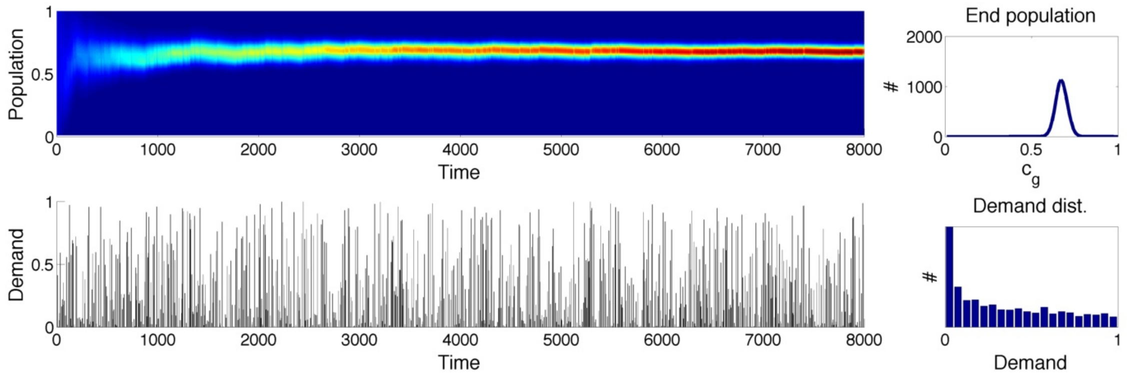

$\lambda = 10, p = 5$

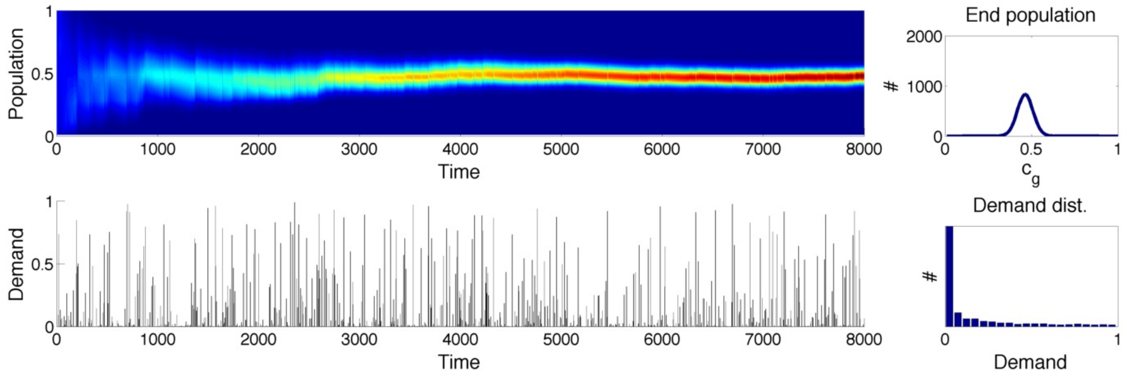

$\lambda = 10, p = 10$

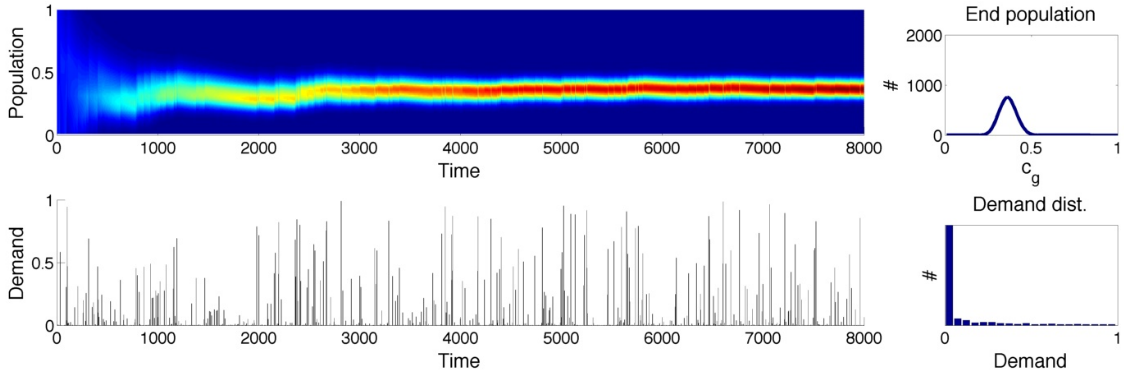

Changing mean time between bursts:

Longer intervals between bursts provide the low-capacity population more time to recover and take over because their growth rate is higher (lower fixed cost).

$\lambda = 5, p = 2$

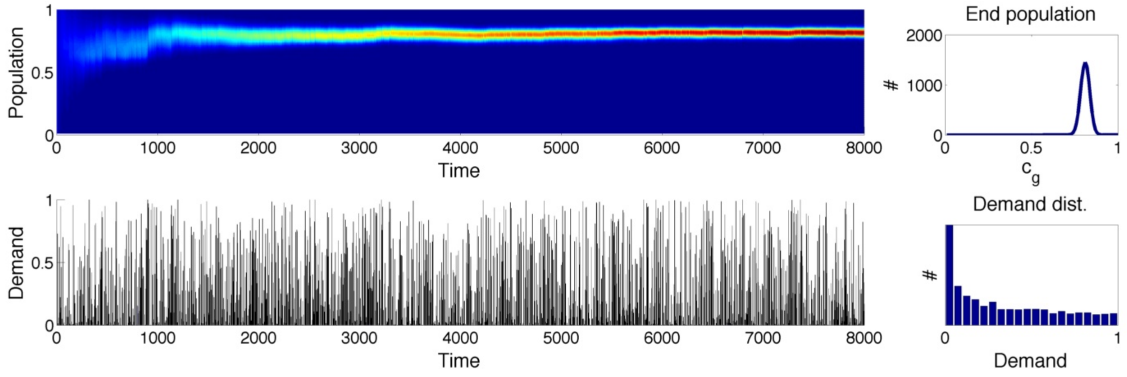

$\lambda = 10, p = 2$

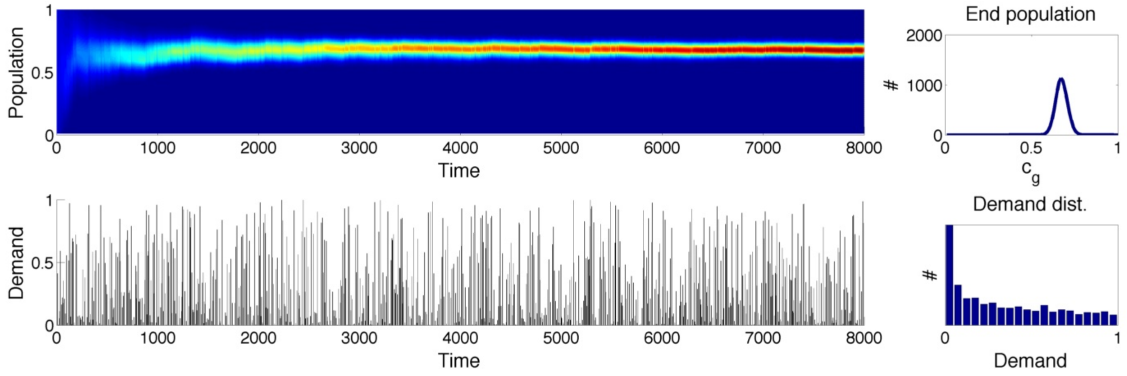

$\lambda = 15, p = 2$

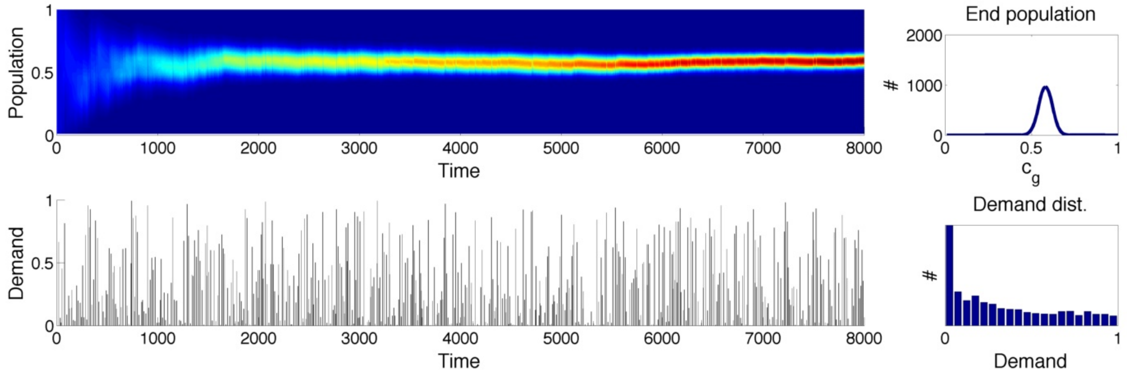

$\lambda = 20, p = 2$

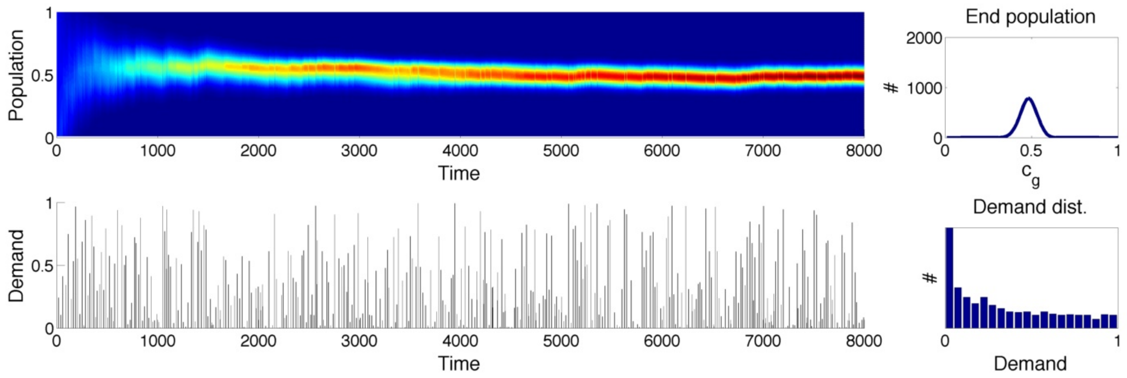
Population
Time
End population
#
c_g
Demand
Time
Demand dist.
#
Demand
